# Depletion of Tip60 from *In Vivo* Cardiomyocytes Increases Myocyte Density, Followed by Cardiac Dysfunction, Myocyte Fallout and Lethality

**DOI:** 10.1371/journal.pone.0164855

**Published:** 2016-10-21

**Authors:** Joseph B. Fisher, Audrey Horst, Tina Wan, Min-Su Kim, John Auchampach, John Lough

**Affiliations:** 1 Department of Cell Biology Neurobiology and Anatomy, and the Cardiovascular Center, Medical College of Wisconsin, Milwaukee, Wisconsin, United States of America; 2 Department of Pharmacology and Toxicology, and the Cardiovascular Center, Medical College of Wisconsin, Milwaukee, Wisconsin, United States of America; Mayo Clinic, UNITED STATES

## Abstract

Tat-interactive protein 60 (Tip60), encoded by the *Kat5* gene, is a member of the MYST family of acetyltransferases. Cancer biology studies have shown that Tip60 induces the DNA damage response, apoptosis, and cell-cycle inhibition. Although Tip60 is expressed in the myocardium, its role in cardiomyocytes (CMs) is unclear. Earlier studies here showed that application of cardiac stress to globally targeted *Kat5*^*+/—*^haploinsufficient mice resulted in inhibition of apoptosis and activation of the CM cell-cycle, despite only modest reduction of Tip60 protein levels. It was therefore of interest to ascertain the effects of specifically and substantially depleting Tip60 from CMs using *Kat5*^*LoxP/-;Myh6-Cre*^ mice in the absence of stress. We report initial findings using this model, in which the effects of specifically depleting Tip60 protein from ventricular CMs, beginning at early neonatal stages, were assessed in 2–12 week-old mice. Although 5’-bromodeoxyuridine immunostaining indicated that CM proliferation was not altered at any of these stages, CM density was increased in 2 week-old ventricles, which persisted in 4 week-old hearts when TUNEL staining revealed inhibition of apoptosis. By week 4, levels of connexin-43 were depleted, and its patterning was dysmorphic, concomitant with an increase in cardiac hypertrophy marker expression and interstitial fibrosis. This was followed by systolic dysfunction at 8 weeks, after which extensive apoptosis and CM fallout occurred, followed by lethality as mice approached 12 weeks of age. In summary, chronic depletion of Tip60 from the ventricular myocardium beginning at early stages of neonatal heart development causes CM death after 8 weeks; hence, Tip60 protein has a crucial function in the heart.

## Introduction

Tip60 (Tat-interactive protein 60 kD) is an acetylase protein, which in cancer cells has been shown to induce the DNA Damage Response (DDR) and apoptosis by respectively acetylating ATM [1,2] and p53 [3,4]. Tip60 has also been shown to inhibit the cell-cycle [5]. These functions aren’t mutually exclusive. Tip60 is encoded by the *Lysine acetyltransferase-5* (*Kat5*) gene. We previously reported that Tip60 is a vital intracellular protein, since ablation of both *Kat5* alleles causes embryolethality at the blastocyst stage of development. However, global heterozygous *Kat5*^*+/-*^ mice reproduce normally, exhibiting no apparent haploinsufficient phenotype under normal conditions [6]. This laboratory wishes to elucidate Tip60’s role in the adult heart, wherein it is relatively enriched [7]. We previously subjected *Kat5*^*+/-*^ heterozygous adult hearts to stress of cMyc over-expression, and to pressure overload [8]. In accord with Tip60’s role in cancer cells, both stressors reduced apoptosis while inducing re-entry of ventricular cardiomyocytes (CMs) into the cell-cycle, despite the retention of Tip60 protein at ~80% of wild-type levels [9].

According to the TSGene database (http://bioinfo.mc.vanderbilt.edu/TSGene/index.html), Tip60 is a tumor suppressor. Because our previous work indicated that modest Tip60 reduction in combination with stress relieved inhibition of the CM cell-cycle [9], similar to the effects of depleting retinoblastoma [10], hippo pathway components [11], and Meis1 [12], it was of interest to ascertain the effects of substantially depleting Tip60 in CM-specific fashion from the mouse heart, in the absence of stress. Hence, we prepared a genetic model wherein most of the *Kat5* coding region, including the Tip60 acetylase domain, is efficiently and specifically disrupted in mouse CMs *in vivo*.

This is our first report using this model, wherein we describe the phenotype caused by CM-specific Tip60 depletion, beginning in the ventricles at early neonatal stages consequent to activation of a constitutively expressed *Myh6*-driven *Cre-recombinase* transgene, in 2, 4, 8 and 12 week-old mouse hearts. Proliferation of ventricular CMs was not altered at any age. However, an increase in CM density was observed in 2 week-old hearts, which persisted through week 4, when TUNEL staining indicated a transient reduction in apoptosis. By week 4, signs of deterioration were also observed, including connexin-43 depletion, increased hypertrophy marker expression, and interstitial myocardial fibrosis. By week 8 these changes became pronounced, leading to significantly impaired cardiac function that culminated in death of most Tip60-depleted mice prior to week 12, when extensive apoptosis and CM fallout were observed. Taken together, these findings indicate that although depletion of Tip60 from the ventricular myocardium may transiently permit CM expansion, its chronic depletion is incompatible with CM survival.

## Materials and Methods

### Animal Care and Use

This investigation adhered to the National Institutes of Health (NIH) Guide for the Care and Use of Laboratory Animals (NIH Pub. Nos. 85–23, Revised 1996). All protocols described in the authors’ Animal Use Application (AUA #000225), which were approved by the Medical College of Wisconsin’s Institutional Animal Care and Use Committee (IACUC), were adhered to in this study. The Medical College of Wisconsin IACUC has an Animal Welfare Assurance status from the Office of Laboratory Welfare (A3102-01). In this study, we observed that Tip60 depletion caused mortality between 8–12 weeks of age. Because this outcome was not anticipated, it was not reviewed and approved by the animal ethics committee as part of our IACUC-approved protocol. Despite daily monitoring to identify humane endpoints based on BCS (body condition scoring, [13]) criteria, neither a BCS score of “3” in a single category nor an aggregate score of “7” across categories was observed prior to death. All male mice (approximately 25 in this study) bearing the *Kat5*^*LoxP/-;Myh6-Cre*^ genotype spontaneously died at approximately 12 weeks of age. [Note: BCS criteria that were monitored included discomfort (assessed by hunching with disheveled fur), lethargy, rapid weight loss, labored breathing, and behavioral response to external stimuli.]. For details see Supporting Information (S1 File).

### Generation of Mice with LoxP-Flanked *Kat5* Alleles

The generation of mice with a globally-targeted (i.e. null) *Kat5* allele, wherein the promoter and exons 1–9 of the *Kat5* gene are replaced with a neomycin resistance gene [14], was described previously [6].

To generate mice with LoxP-flanked *Kat5* alleles, a targeting vector was prepared by recombineering [15,16], resulting in the insertion of LoxP sites into introns 2 and 11 of the *Kat5* gene (S1A Fig). Correctly targeted ESCs (line V6.5) were injected into C57/Bl6 blastocysts, which implanted and transmitted the targeted allele, termed *Kat5*^*LoxP(FRTneoFRT)/+*^, via germline. A *neomycin phosphotransferase* (*neo*) cassette in intron 2 was subsequently removed by mating with a mouse expressing a *Flp recombinase* transgene, resulting in the genotype *Kat5*^*LoxP/+*^, which was then bred to homozygosity (*Kat5*^*LoxP/LoxP*^). Genotyping performed by PCR and Southern blotting confirmed targeting accuracy (S1B and S1C Fig).

Mice containing the cardiac-specific *Myh6*-driven *Cre-recombinase* transgene, which is briefly activated during early development followed by strong and persistent expression in the ventricles beginning at early neonatal stages [17], were purchased from the Jackson Laboratory (*Tg(Myh6-cre)1Jmk/J*; #009074). This transgene was bred into the heterozygous *Kat5*^*+/-*^ background, then backcrossed to produce a stud line that was heterozygous for *Kat5* and homozygous for *Myh6-Cre* (i.e. *Kat5*^*+/-;Myh6-Cre/Myh6-Cre*^). The latter were bred with *Kat5*^*LoxP/LoxP*^ mice to obtain the experimental (*Kat5*^*LoxP/-;Myh6-Cre*^) and control (*Kat5*^*LoxP/+;Myh6-Cre*^) genotypes that were compared in this study. The experimental genotype (*Kat5*^*LoxP/-;Myh6-Cre*^) was chosen to increase efficiency of Tip60 protein removal, because only one *Kat5* allele had to be ablated, and since our previous work revealed that *Kat5*^*+/-*^ heterozygous mice (i) reproduce normally, (ii) have no apparent adult phenotype and (iii) express near-normal levels of Tip60 protein [9]. Although wild-type mice with either one or two copies of the *Myh6-Cre* transgene exhibit no phenotype [18], breeding was adjusted so that only one copy was inherited. Because the *Myh6-Cre* transgene is located on the X-chromosome, the extent of Tip60 depletion occurs in gender-specific fashion (S2A–S2C Fig), *Myh6-Cre* is expressed in all CMs in male hearts, but in only one-half of the CMs in female hearts. Hence, only male mice were used in these experiments (with the exception of the echocardiography determinations; S2D and S2E Fig).

### Echocardiography

Echocardiography was performed as described in Supporting Information (S1 File).

### Genotyping

Genotyping was performed by PCR in 25.0 μl reactions that included 1x GoTaq G2 Green Mastermix (Promega #M7822), 0.5 μM each primer, and 4.0 μl template. Templates consisted of low-speed supernatants of extracted ear tissue samples (punches) that had been boiled for 10 minutes in 0.3 ml 10 mM NaOH/1 mM EDTA. Primer pairs used to amplify each allele are listed in Table 1. PCR products were amplified in an AB Applied Biosystems GeneAmp PCR System 9700 followed by separation and imaging of amplicons on ethidium bromide-stained 1% agarose gels.

**Table 1:** Primer Pairs.

for PCR Genotyping						
Allele	Sequence (5’-3’) and Working Conc.			Lough lab Identifier	Amplicon (bp)	Annealing °C

Null (-)	FWD	GACAGACTCGGCGTTCCTCCAATC	0.75 μM	5057F	278	55
	REV	GGCCAGCTCATTCCTCCACTCATGATC	0.15 μM	Neo1621		
Wild-Type (+)	FWD	GACAGACTCGGCGTTCCTCCAATC	0.75 μM	5057F	379	
	REV	CGGCAGCCCTCGATTATCTC	2.25 μM	5436R		
Cycling Details: 95°C 5 min, then 35 cycles of 95°C 60sec/55°C 60sec/72°C 60sec, then 72°C 7 min. The above two alleles can be amplified independently, or they can be amplified in multiplex by mixing primers 5057F, Neo1621 and 5436R at the indicated working concentrations.						

LoxP in intron 2	FWD	AGGGAGTCAACGATCGCACGGGAGG	0.5 μM	LFNF-fwd	359 LoxP 258 WT	67
	REV	CACAGACAGGGAGTCTTAGCCAGGG	0.5 μM	LFNF-rev		
Cycling Details: 94°C 1 min, then 35 cycles of 94°C 45sec/67°C 35sec/72°C 60sec, then 72°C 10 min. Both primers anneal to intron 2 of the *Kat5* gene. Neither of these primers anneals to the null allele of the global knockout mice because intron 2 is ablated and replaced by the neomycin resistance gene.						

LoxP in intron 11	FWD	CTGTGTCTTCTGGCCAAGTGTT	0.5 μM	d813	785 LoxP 684 WT	56
	REV	TCGGTTCTCAGAGACTAGC	0.5 μM	96c		
Cycling Details: 94°C 3 min, then 35 cycles of 94°C 45sec/56°C 35sec/72°C 60sec, then 72°C 10 min						

*Myh6-Cre t*ransgene	FWD	GCGGTCTGGCAGTAAAAACTATC	1.0 μM	per Jackson Lab #009074	~100	52
	REV	GTGAAACAGCATTGCTGTCACTT	1.0 μM			
Cycling Details: Cycling Details: 94°C 3 min, then 35 cycles of 94°C 30sec/52°C 60sec/72°C 60sec, then 72°C 2 min						

**for Assessing Recombination of the *Kat5* Allele**						
Recombined LoxP *Kat5* Allele	FWD	AGGGAGTCAACGATCGCACGGGAGG	0.64 μM	LFNF-fwd	444 recomb. 5,020 intact	58
	REV	TCGGTTCTCAGAGACTAGC	0.64 μM	96c		
Cycling Details: 94°C 3 min, then 40 cycles of 94°C 45sec/58°C 35sec/72°C 60sec, then 72°C 10 min; additional cycles can be run using an annealing temperature of 64°C.						

### Assessment of Cre-Mediated *Kat5* Recombination

Cre-mediated recombination of the *Kat5* allele was assessed by PCR on samples of left ventricle (LV) and liver, using the primer pair listed in Table 1. These primers, which anneal to introns 2 and 11, amplify 444 and 4,793 bp segments in the presence and absence of recombination, respectively.

### Heart Tissue Processing

This was performed by anesthetizing mice with 4% isoflurane, followed by removal of the anterior chest wall and rapidly perfusing the heart by gravity with 20 ml ice-cold cardioplegic solution (25 mM KCl/5% dextrose/ PBS) introduced via the left ventricular apex with a 27-gauge needle. Hearts were rapidly removed and the LV was isolated, followed by transverse sectioning to obtain superior and inferior parcels that were respectively used for western blotting/mRNA analysis and immunostaining.

### Quantitative RT-PCR (qPCR)

All qPCR reactions were performed per the manufacturers’ recommendations. Heart tissue was disrupted with a Tissue Lyser II and total RNA was purified using the RNeasy Plus Mini Kit (Qiagen #74134) followed by quantitation using a Qubit Fluorometer Broad Range RNA quantification device. First-strand cDNA was synthesized by reverse-transcribing 1.0 μg total RNA using iScript (Bio-Rad #170–8890). PCR was performed by adding 5% of the reverse transcription product as template to SYBR Green Supermix (BioRad #1725260) that included 1.5 mM MgCl_2_, 200 μM of each dNTP and 0.5 μM of each primer. Reactions were performed in 20 μl total volume using PrimePCR Custom 96-well plates (Bio-Rad #100–25216) embedded with proprietary Bio-Rad primers.

### Western Blotting

Blots were prepared using total protein extracted from the superior half of the left ventricle, which was minced with scissors, suspended in lysis buffer (20mM HEPES [pH 7.5]/150mM NaCl/1mM EDTA/100mM dithiothreitol/1% Triton-X-100/5% glycerol plus 1x protease inhibitor (Roche #1836153) and phosphatase inhibitor cocktail) and sonicated. Protein concentration was determined using the Bradford assay (Bio-Rad #500–0006). Samples were stored at -80°C until electrophoresis. For electrophoresis, samples containing 10 μg total protein were suspended in SDS sample buffer (250 mM Tris-HCl [pH 6.8]/3.75% glycerol/0.005% bromphenol blue/2% SDS/100mM dithiothreitol), heated at 100°C for 5 minutes, and electrophoretically separated on 7.5% acrylamide:bis (30:0.8)/SDS gels (Bio-Rad #161–1118). Separated proteins were electroblotted onto nitrocellulose membrane (Bio-Rad #162–0146) and blocked overnight with NFDM/TBST (5% non-fat dry milk/10mM Tris-HCl (pH 7.6)/150mM NaCl/0.05% Tween-20).

Primary and secondary antibodies and dilutions are listed in Table 2. Blots were reacted with primary antibody in blocking buffer overnight at 4°C. The affinity-pure anti-Tip60 was custom prepared (Bethyl Labs.) in rabbit, using the N-terminal peptide EGCRLPVLRRNQDNE peptide as immunogen. This peptide is present in the N-terminus of all Tip60 isoproteins and is absent from all other proteins including the highly related MYST family member MOF. This antibody was characterized as previously described in detail [9]. Tip60 blots were simultaneously reacted with anti-GAPDH. Secondary antibodies were diluted in NFDM/TBST and applied for 60 minutes at RT. Blots were then covered with HRP-substrate (Amersham #RPN2232) for 1 minute at room temperature followed by antigen localization, which was accomplished by exposing the membrane to Hyperfilm-ECL (Kodak #8294985) for selected intervals up to 20 minutes and processing in a Kodak X-Omat 2000A developer. Films were scanned with a Hewlitt-Packard Scanjet G4010 scanner; densitometry was quantified using ImageJ software.

**Table 2:** Antibodies.

	for Immunofluorescence (IF)				
	Antigen	Manufacturer	Catalog#	Made in	Dilution
1°	5’-bromodeoxyuridine	Abcam	ab1893	sheep	1:75
2°	donkey anti-sheep 488	Invitrogen	A-11015	donkey	1:500

1°	cre-recombinase	EMD-Millipore	69060–3	rabbit	1:3000
2°	goat anti-rabbit 594	Invitrogen	A-11037	goat	1:500

1°	connexin-43	Santa Cruz	sc-9059	rabbit	1:50
2°	goat anti-rabbit 488	Invitrogen	A-11008	goat	1:500

	**for Immunohistochemistry (IHC)**				
1°	caspase-3	Biocare	CP229	rabbit	1:100
2°	goat anti-rabbit, biotin-SP	Jackson ImmunoRes	111-065-003	goat	1:1000
3°	peroxidase streptavidin	Jackson ImmunoRes	016-030-084		

	**for Western Blotting**				
1°	Tip60	Bethyl	custom	rabbit	1:1000
2°	goat anti-rabbit IgG HRP	Bio-Rad	170–6515	goat	1:7500

1°	GAPDH	Adv ImmunoChem	2-RGM2	mouse	1:1000
2°	goat anti-mouse IgG HRP	Bio-Rad	170–6516	goat	1:7500

1°	connexin-43	Santa Cruz	sc-9059	rabbit	1:100
2°	goat anti-rabbit IgG HRP	Bio-Rad	170–6515	goat	1:7500

### Immunostaining & Cell Counting

Two days before harvest, mice were intraperitoneally injected with 5’-bromodeoxyuridine (1.0 mg/100 μl; Sigma #B9285). Perfused hearts were fixed overnight in fresh 4% paraformaldehyde/PBS, processed through 70% EtOH, and embedded in paraffin. Sections (5 μm) on microscope slides were immunostained using the primary and secondary antibodies listed in Table 2. Cells were manually counted by scanning the entirety of each section at 600x magnification. BrdU-labeled cells were scored as positive only if stain was clearly confined to the nucleus (verified with DAPI), and was granular. Caspase-3-labeled cells were scored as positive only if clearly defined granular immunohistochemical product was observed in the cytoplasm. Cells at edges of sections were not scored, in order to avoid including edge-artifacts. Due to their large numbers, CM nuclei, which were efficiently identified using Cre-recombinase immunostaining, were enumerated by photographing six 400x fields in each section, followed by ImageJ particle analysis. Briefly, using ImageJ software, the red (594) channel containing Cre-recombinase-positive nuclei was isolated and smoothened 4x. This was followed by respectively adjusting brightness:contrast to 30:35. Particles (red-stained nuclei) were then analyzed by setting the range of pixel sizes to 250-infinity, and of circularity to 0.5–1.0. Numbers and average sizes of particles (red-stained nuclei) were recorded. Cells at the periphery of sections, including the epicardium, were excluded during both manual and automated counting.

### TUNEL Labeling & Counting

Apoptosis was assessed using the DeadEnd Fluorometric TUNEL System (Promega #G3250) per the manufacturer’s recommendations. Total numbers of TUNEL-positive nuclei in each section were manually counted at 400x magnification. TUNEL signal was counted only if it was confined to a DAPI-positive nucleus. Adherence to this convention likely underestimated total numbers of TUNEL-positive nuclei in 8 and 12 week-old hearts wherein nuclei were deteriorating. Nuclei were scored as TUNEL-positive only if at least 50% of the nucleus was filled with signal. Within each heart, 1,500–5,000 cells were enumerated to calculate percentages of labeled cells, relative to total numbers of DAPI-stained nuclei that were counted by automated scanning.

## Results

The initial objective of this study was to compare the effects of depleting Tip60 from CMs in the left ventricle of experimental mice having the *Kat5*^*LoxP/-;Myh6-Cre*^ genotype, with control mice having the *Kat5*^*LoxP/+;Myh6-Cre*^ genotype, at 4, 8 and 12 weeks of age. These genotypes were selected because the only genetic variable was the presence or absence of the null allele; hence, the effects, if any, of Cre protein [19] were self-canceling. To increase efficiency of Tip60 depletion, experimental mice were bred to contain a null *Kat5* allele instead of a second LoxP-flanked allele, since only one *Kat5* allele had to be recombined, and because our previous work had revealed that *Kat5*^*+/-*^ heterozygous mice express Tip60 protein at near wild-type levels [9]. Breeding was adjusted so that only one copy of the *Myh6-Cre* transgene was present. Because this transgene is located on the X-chromosome, causing Tip60 to be depleted in gender-specific fashion, only male mice were evaluated except as shown in S2 Fig. All assessments were restricted to the ventricular myocardium.

**Fig 1:**
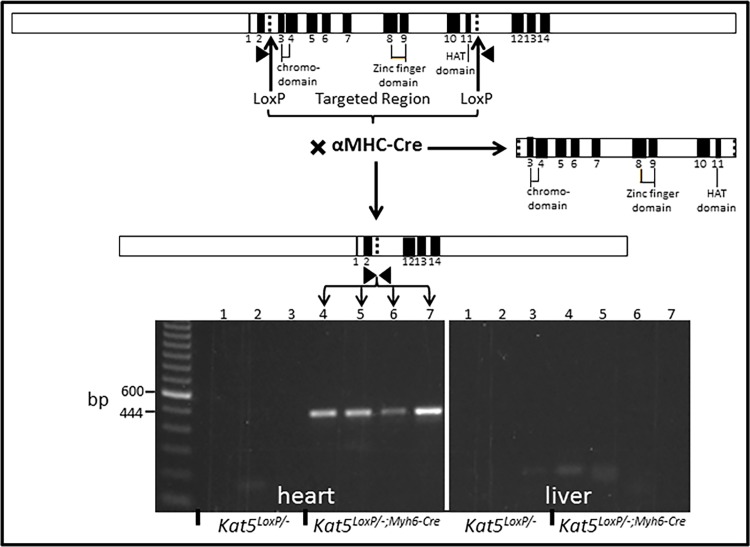
αMHC-Cre Disrupts the LoxP-flanked *Kat5* (Tip60) Gene. **Above:** restriction map of LoxP-targeted allele; arrowheads denote primer sites. Middle: map of re-combined allele in *Kat5*^*LoxP/-;Myh6-Cre*^ adult male mice. **Below:** 444 bp PCR product amplified from 4 week-old male mice, indicative of CM recombination because this amplicon is see only in heart, not liver. Each lane contains genomic DNA amplified from heart or liver tissue of a different animal.

The LoxP sites located in introns 2 and 11 of the *Kat5* gene (Fig 1, upper) were designed to undergo recombination induced by Cre-recombinase, the *Myh6-Cre* driver for which becomes strongly expressed in the ventricles near the time of birth [17]. This resulted in ablation of approximately two-thirds of the coding region, including Tip60’s chromo and acetylase domains (Fig 1, right). As shown in Fig 1, PCR amplification of the *Kat5* allele in *Kat5*^*LoxP/-;Myh6-Cre*^ mice revealed that the LoxP-targeted allele was disrupted in tissue-specific fashion, as revealed by the 444 bp product indicative of recombination in the heart, which was not detected in liver samples (Fig 1, lower).

**Fig 2:**
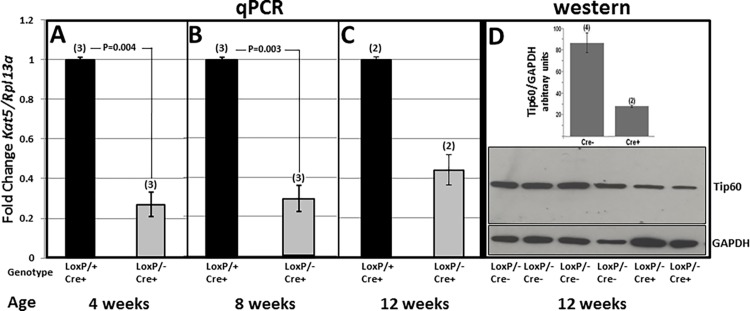
Disruption of *Kat5* Reduces Levels of Tip60 Protein and mRNA. **Panels A-C** show qPCR determinations revealing reduced levels of *Kat5* mRNA in hearts of 4, 8 and 12 week-old male *Kat5*^*LoxP/-;Myh6-Cre*^ mice; in **C**, because few *Kat5*^*LoxP/-;Myh6-Cre*^ male mice survived until 12 weeks, numbers of viable hearts were limited. The western blot (**D)** compares Tip60 protein levels in hearts of four *Kat5*^*LoxP/-*^ (i.e. *LoxP/-;Cre-*) mice with two *Kat5*^*LoxP/-;Myh6-Cre*^ (i.e. *LoxP/-;Cre+*) mice; these respective control and experimental phenotypes were used in this determination due to limited numbers of the latter at the 12 week-old timepoint. P-values were calculated using Student’s T-Test (two-tailed).

The effect of *Kat5* disruption on Tip60 mRNA and protein levels was respectively evaluated by RT-PCR at weeks 4, 8 and 12, and by western blotting at week 12. The former revealed that mRNA levels were reduced by approximately 75% at 4 and 8 weeks, followed by a less extensive decrease at 12 weeks (Fig 2A–2C). Tip60 protein levels relative to GAPDH were similarly depleted in 12 week-old *Kat5*^*LoxP/-;Myh6-Cre*^ hearts (Fig 2D). Note in Fig 2D that the normalized level of Tip60 protein depletion in 12 week-old *Kat5*^*LoxP/-;Myh6-Cre*^ hearts is likely over-estimated due increased GAPDH levels, which may reflect glycolytic/hypoxic changes or more likely the ingression of fibroblasts and inflammatory cells into the myocardium; this may also explain the apparent increase in mRNA levels in these hearts. Importantly, Tip60 levels were more extensively reduced in *Kat5*^*LoxP/-;Myh6-Cre*^ hearts (Fig 2D) than in Tip60-haploinsufficient (*Kat5*^*+/-*^) hearts [9], wherein Tip60 protein was maintained at near-normal levels. Finally, it is emphasized that because CMs constitute a minority cell type in the murine heart [20,21], complete depletion of Tip60 in these heterogeneous tissue samples was not anticipated, despite the complete depletion of cardiomyocytes from *Kat5*^*LoxP/-;Myh6-Cre*^ hearts described below.

**Fig 3:**
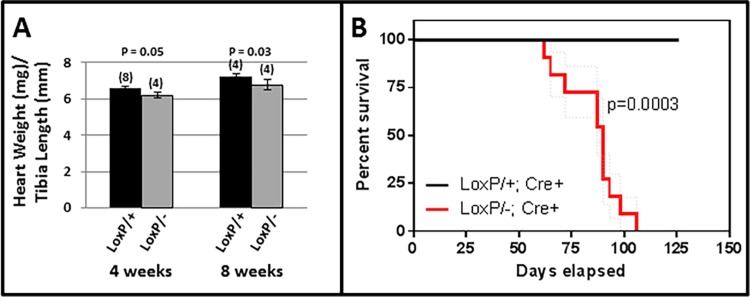
Tip60 depletion causes reduced heart mass and diminished survival. **A**, Heart mass is slightly reduced in *Kat5*^*LoxP/-;Myh6-Cre*^ hearts at 4 and 8 weeks of age. **B**, Survival of mice bearing the *Kat5*^*LoxP/+;Myh6-Cre*^ and *Kat5*^*LoxP/-;Myh6-Cre*^ genotypes was compared with Kaplan-Meier statistics using Prism software, which uses the method of Greenwood to calculate standard errors. The P-value was calculated using two-tailed Chi-square analysis (1 DF).

**Fig 4:**
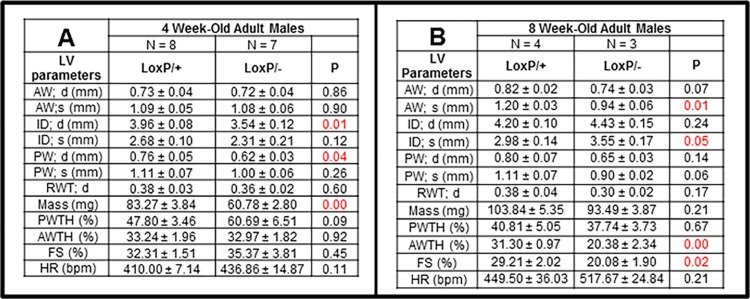
Reduced Cardiac Function in Tip60-depleted Male Hearts. In *Kat5*^*LoxP/-;Myh6-Cre*^ male mice, cardiac dysfunction revealed by reduced fractional shortening (FS) occurs between 4 (**A**) and 8-weeks of age (**B**). P-values were calculated using Student’s T-Test (two-tailed, unpaired).

As shown in Fig 3A, hearts in Tip60-depleted male mice were slightly diminished in size by 4 and 8 weeks of age. Remarkably, as shown in Fig 3B, these mice began to die after the 8 week timepoint, with few survivors remaining by 12 weeks of age. The echocardiographic assessments shown in Fig 4 revealed that although systolic function (%FS) was normal at 4 weeks, heart mass was reduced (Fig 4A), consistent with the assessment of heart weight/tibia length at that age (Fig 3A). By 8 weeks, systolic parameters were altered, culminating in significantly reduced fractional shortening (Fig 4B). Although female mice, in which only one-half of the CMs expressed the *Cre-recombinase* transgene (S2C Fig), survived longer, these exhibited cardiac dysfunction as early as 20 weeks of age (S2D Fig).

**Fig 5:**
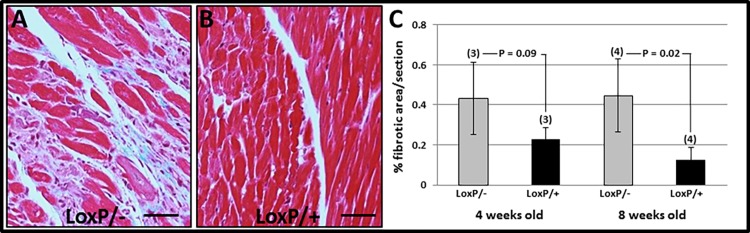
Tip60-Depletion Causes Cardiac Fibrosis. Fibrosis in male hearts was determined by Masson’s Trichrome staining, followed by quantitative scanning of sections to quantitate percentages of fibrotic (blue) area. **A** and **B** are representative fibrotic areas in 8 week-old *Kat5*^*LoxP/-;Myh6-Cre*^ and *Kat5*^*LoxP/+;Myh6-Cre*^ hearts, respectively. **C**, results of quantitative scanning showing extent of fibrosis. All mice contained the *Myh6-Cre recombinase* transgene. N = number of hearts evaluated; error bars = ±SD; size bars = 60 μm. P-values were calculated using Student’s T-Test (two-tailed).

**Fig 6:**
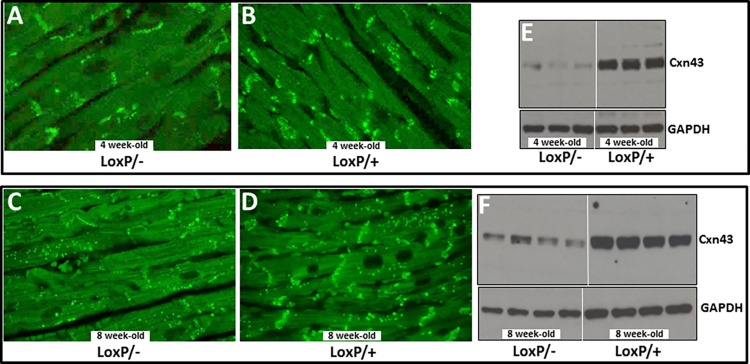
Connexin-43 Depletion in Tip60-Depleted Hearts. Heart samples were immunostained (**A-D**) or western blotted (**E-F**) to detect connexin-43. Panels **A-D** reveal that progressively diminished expression in the intercalated disc transverse component occurs between weeks 4 and 8. The western blots (**E-F**) reveal strong depletion of Cxn43 by 4 weeks. Protein in each lane was from an individual mouse heart. All mice contained the *Myh6-Cre recombinase* transgene.

These changes in Tip60-depleted hearts of male mice were accompanied by increased expression of the pathologic hypertrophy marker atrial natriuretic factor (ANF) at 4 weeks, followed by significantly increased levels of ANF as well as β-myosin heavy chain (β-MHC) at 8 weeks (S3 Fig). Increased levels of interstitial fibrosis were also seen by 4 weeks (Fig 5). Perhaps most remarkably, content of connexin-43 (Cxn43) protein was substantially depleted in 4 and 8 week-old hearts (Fig 6E and 6F); these reductions were accompanied by dysmorphic patterning of Cxn43 expression in Tip60-depleted hearts (Fig 6A–6D).

The results shown in Figs 3–6 demonstrated that CM survival was crucially dependent on the presence of Tip60 protein. To ascertain Tip60’s crucial role in the myocardium, it was decided to assess the effects of Tip60 depletion on CM apoptosis and proliferation at younger ages, beginning with 2 week-old hearts.

**Fig 7:**
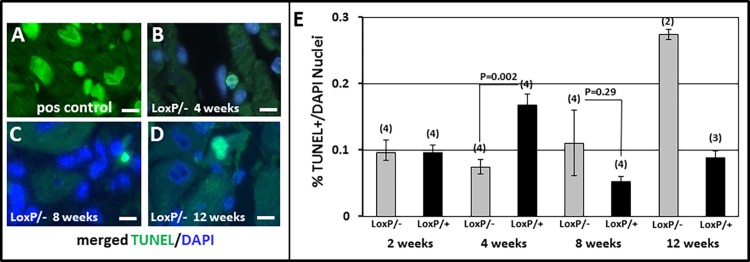
TUNEL Staining in Tip60-depleted Myocardium. **Panels B-D** show TUNEL labeling in *Kat5*^*LoxP/-;Myh6-Cre*^ hearts at the indicated ages. **Panel A** is a positive control (not merged with DAPI) generated by pre-treating *Kat5*^*LoxP/+;Myh6-Cre*^ CMs with DNase1. Size bars in A-D = 10 μm. **Panel E**, average numbers of TUNEL-positive cells in each 400x section, normalized to the total number of DAPI-positive nuclei assessed by automated scanning. Bars = averages. N = number of mouse hearts evaluated, vertical lines = ±SEM. P-values were calculated using Student’s T-Test (two-tailed).

Because Tip60 had previously been shown to induce apoptosis in cancer cells [3,4,22], and more recently in cultured CMs under stress [23], we attempted to assess, despite its normally low levels in non-stressed myocardium, whether the incidence of apoptosis was further reduced by Tip60 depletion. As revealed by TUNEL staining (Fig 7E), apoptosis was not altered in 2 week-old *Kat5*^*LoxP/-;Myh6-Cre*^ hearts. However, at 4 weeks of age, percentages of TUNEL-positive nuclei were significantly and strongly reduced in the left ventricle. Thereafter, as anticipated by the onset of pathogenesis at week 4 (Figs 3–6), a trend towards significantly increased apoptosis was observed. Although caspase-3 immunostaining did not detect the decline in apoptosis revealed by TUNEL at week 4, observations of increased apoptosis at weeks 8 and 12 were corroborated (S4E Fig).

**Fig 8:**
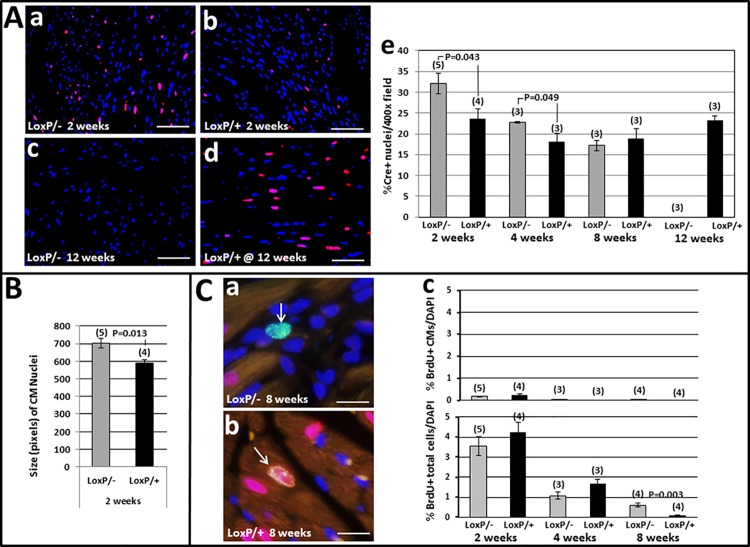
CM Density & Proliferation in Tip60-depleted Myocardium. **Panel A: CM Density. a-d,** immunofluorescent Cre in CM nuclei (red) in comparison with total nuclei (blue, DAPI). Bars = 30 μm. **e,** percentage of Cre-positive nuclei relative to total DAPI-positive nuclei per 400x field determined by ImageJ. **Panel B: Size of CM Nuclei** determined by ImageJ. **Panel C: CM Proliferation. a-b,** double immunofluorescent BrdU (green) and Cre-recombinase (Cre; red) images. **a**, typical BrdU+ non-myocyte nucleus (arrow). **b**, BrdU+Cre double-labeled CM nucleus (arrow). Bars = 10 μm. **c**, upper and lower, percentages of BrdU-positive CMs and total cells, respectively. BrdU-positive nuclei were manually counted and expressed as a percentage of DAPI (blue)-stained nuclei determined by automated scanning. In all panels, bars = averages. N = number of mouse hearts evaluated, error bars = ±SEM. P-values were calculated using Student’s T-Test (two-tailed). All CMs contained the *Myh6-Cre* transgene.

Because Tip60 had previously been shown to inhibit the cell-cycle [4,24,25], we also assessed whether CM proliferation and/or CM density was increased in Tip60-depleted cells (Fig 8). Increased proliferation of total myocardial cells (5’-BrdU-positive cells in Fig 8Cc, lower), most of which were presumably fibroblasts (consistent with fibrosis; Fig 5) and/or inflammatory cells, was detected in 8 week-old Tip60-depleted hearts. Although increased CM proliferation was not observed at any timepoint (Fig 8Cc, upper), significantly increased CM density was seen in both 2 and 4 week-old Tip60-depleted hearts (Fig 8Ae). It may also be noteworthy that CM nuclei were significantly enlarged in 2 week-old Tip60-depleted hearts (Fig 8B). By 8 weeks, CM density was unchanged. Remarkably, this was followed by the complete fallout of CMs from Tip60-depleted hearts by 12 weeks of age (Fig 8Ae).

## Discussion

Tip60, a pleiotropic protein, has been shown to induce the DNA damage response (DDR [2,26]), apoptosis [3,4], and cell-cycle regulation [5] in cancer cells. Previous work performed here [9] and in another laboratory [23] has indicated that Tip60 may have similar functions in cardiomyocytes (CMs). However, the effect of specifically and extensively removing Tip60 from *in vivo* CMs in the absence of stress remained unknown. This report describes initial experiments designed to address this question. Our findings indicate that protracted depletion of Tip60 from CMs in the *in vivo* left ventricle from the time of birth is incompatible with survival, resulting in complete CM fallout (Fig 8Ae), and death (Fig 3B) of the animal, between 8–12 weeks of age. Although this study focused on ventricular changes, it must also be considered that depletion of Tip60 from CMs in the developing atria, which is anticipated when employing the *Myh6*-driven cre transgene [18], may have contributed to the ventricular phenotype; for example it has been shown that mutations that specifically disrupt the developing atria also affect form and function in the developing ventricle [27,28]. In any event, however, our results show that as in pluripotent cells of the blastocyst [6], Tip60 is necessary to maintain survival of differentiated CMs.

We previously addressed this question using Tip60 heterozygous (*Kat5*^*+/-*^) mice, wherein a single *Kat5* allele was disrupted in all cells (i.e. global ablation). Unlike *Kat5*^*-/-*^ mice which die as blastocysts [6], *Kat5*^*+/-*^ mice maintain Tip60 protein at near-normal levels while exhibiting no reproductive or haploinsufficient defects at any stage [9]. To elicit a haploinsufficient response in the heart, stress was applied by over-expressing c-Myc (to induce the cell-cycle [10]) or by trans-aortic constriction (to induce CM hypertrophy). This resulted in cell-cycle activation and reduced apoptosis [9], consistent with Tip60’s role in cancer cells. In the current study, with the exception of reduced TUNEL staining observed in 4 week-old hearts (Fig 7E), which was not corroborated by caspase-3 staining (S4 Fig), these effects were not phenocopied. We speculate that this indicates that the lethal effects of protracted and extensive Tip60 depletion, which culminate in complete CM fallout and death by 12 weeks (Figs 8Ae and 3B), overshadowed responses reflecting Tip60’s function in CMs at all the stages examined here, even as early as 2 weeks. We further speculate that effects supporting the hypothesis, such as extended cell-cycle transit, may have transiently occurred at early neonatal stages that were not examined in this study. The latter possibility is consistent with the observation of increased CM density in 2 and 4 week-old hearts (Fig 8Ae).

The identification of endogenous factors that maintain CMs in proliferative senescence is currently of high interest (reviewed in [29]). To date, tumor suppressors including retinoblastoma [10] and components of the Hippo pathway [11], as well as Meis1 [30], have been implicated in CM cell-cycle inhibition. Whether Tip60, which has been accorded tumor suppressor status [31], is among these factors remains unresolved. Identification of these factors may be hampered by the CM’s evolution of highly differentiated cellular architecture to accommodate cardiac function, including sarcomeres and intercalated discs, since recent evidence indicates that cell-cycle re-activation requires their de-differentiation ([32,33], reviewed in [34]). A major component of the intercalated disc, connexin-43, which has been shown to inhibit CM proliferation [35,36], becomes strongly depleted several weeks prior to end-stage heart failure in afflictions ranging from myocarditis to ischemic heart disease (reviewed in [37]). It is therefore interesting to consider the possibility that the marked reduction of connexin-43 in heart disease, as well as during Tip60 depletion (Fig 6), indicates an abortive attempt of CMs to re-enter the cell-cycle in order to mediate cardiac regeneration.

It was recently reported that the transition from an hypoxic to a normoxic environment at the time of birth causes oxidative DNA damage in CMs, in response to which the DDR is initiated, which in turn causes CM replicative senescence [38]. In that study, suppression of DNA damage until P7 by maintaining mice in an hypoxic environment, or by scavenging reactive oxygen species (ROS), increased neonatal CM proliferation; the latter treatment also caused a significant increase in CM numbers by P14, similar to the increase in CM density in Tip60-depleted hearts we observed at the same age (Fig 8Ae). Hence it is of extraordinary interest that a wealth of accumulating evidence now exists showing that cells initially respond to DNA damage by activating Tip60, which in turn induces the DDR by acetylating targets including ATM and histone H2AX [1,2,26]. It will therefore be of considerable interest to assess whether, at the time of birth, Tip60 responds to oxidative DNA damage by initiating the DDR, thereby inducing arrest of the CM cell-cycle.

In summary, these results demonstrate that Tip60 protein is necessary for long-term CM vitality and survival. It nonetheless remains possible that transient, less exhaustive depletion of this crucial protein from CMs permit cell-cycle transit while reducing apoptosis. We are addressing these possibilities at early neonatal stages using this model, and at adult stages following acute depletion of Tip60 from tamoxifen-treated *Kat5*^*LoxP/LoxP;Myh6-MerCreMer*^ mice.

## Supporting Information

S1 Fig Targeting the *Kat5* (*Tip60*) Gene. The targeting vector (**Panel A**) was prepared by recombineering to introduce LoxP sites into introns 2 and 11 of the *Kat5* gene. This permits Cre-recombinase-mediated excision of exons 3–11, which consists of 71% of the exon structure including the acetyltransferase domain. Correctly targeted ESCs (line V6.5) were injected into C57Bl6 blastocysts, generating 100% chimeric males that transmitted the floxed allele via germline, providing the genotype *Kat5*^*LoxP(FRTneoFRT)/+*^. The neomycin phosphotransferase (neo) gene in intron 2, which was flanked by FRT sites, was removed by mating with a mouse expressing the Flp recombinase transgene. *Kat5*^*LoxP/-*^ mice, obtained by mating *Kat5*^*LoxP/LoxP*^ mice with *Kat5*^*+/-*^ mice, were used for all experiments reported here because this necessitates deletion of only one allele to maximally reduce Tip60 protein levels. **Panel A, Restriction Map** of the LoxP-targeted *Kat5* allele. Bars denote Southern blot probes A-D. Arrowheads denote PCR primer sites. **Panel B, PCR Genotyping:** The primer pair denoted by green arrowheads (A) amplifies a 250 bp band of the WT allele, which does not contain the previously removed *Neo* cassette. The 5’ green + 3’ blue (in *neo*) arrowhead pair in A amplifies 580 bp of the LoxP-modified allele. The red-black arrowhead pair amplifies 2,301 bp of the LoxP-modified allele (note that the red primer anneals to the LoxP locus). **Panel C, Southern Blot Genotyping. a**, double digest with Hind III (H3) and XhoI (within the LoxP site) followed by hybridization with probe B reveals a 3391 band diagnostic of the floxed allele, and a 7,587 band diagnostic of the WT allele; **b**, digestion with KpnI and hybridization with probe C yields a 7270 bp band from the WT allele, and a 6362 bp fragment created by a KpnI site within the *neo* cassette of the floxed allele; **c** is the same as b except that hybridization was with probe A, an internal probe used to ensure that the targeting vector did not randomly insert into a non-endogenous target(s).
(TIF)

S2 Fig Reduced Cardiac Function in Tip60-depleted Female Hearts. **A-C**, typical immunohistochemical staining pattern of Cre-recombinase showing labeled nuclei in approximately one-half of female (**C**), and in 100% of male (**B**) CMs. This phenomenon is presumably caused by presence of the *Myh6-Cre* transgene on the X chromosome, half of which randomly undergo X-inactivation in female mice. **D-E**, Echocardiographic determinations performed on female hearts showing that although survival is not compromised up to 34 weeks of age, systolic function is affected by week 20. P-values were calculated using Student’s T-Test (two-tailed; unpaired). All CMs contained the *Myh6-Cre* transgene.
(TIF)

S3 Fig Tip60-Depleted Adult Hearts Exhibit Increased Hypertrophy Marker Expression. qPCR showing progressively increased expression of ANF and β-MHC in Tip60-depleted hearts of 4 and 8 week-old male mice. P-values were calculated using Student’s T-Test (two-tailed). All CMs contained the *Myh6-Cre* transgene.
(TIF)

S4 Fig Increased Caspase-3 Staining in Tip60-Depleted Myocardium. Cells in panels **A-D** were photographed at 60x, revealing cytoplasmic granular staining of caspase-3 (Biocare cp229b) in *Kat5*^*LoxP/-;Myh6-Cre*^ (F/-;cre) hearts at each indicated age. Panel **A** is a positive control (mouse lymph node). In **E**, entire sections from each heart were scanned at 60x, during which all caspase-3-positive cells in each section were manually enumerated, followed by normalization to the total number of nuclei assessed by automated scanning of DAPI-stained immediately adjacent sections. F/-;cre denotes hearts from *Kat5*^*LoxP/-;Myh6-Cre*^ mice; F/+;cre denotes hearts from *Kat5*^*LoxP/+;Myh6-Cre*^ mice. The number above each bar indicates the number of mouse hearts that were evaluated. Vertical lines = ±SEM. P-values were calculated using Student’s T-Test (two-tailed). All CMs contained the *Myh6-Cre* transgene.
(TIF)

S1 File Supporting Information. Details regarding animal care and echocardiography.
(DOCX)

## References

[1] Sun Y, Jiang X, Price BD (2010) Tip60: connecting chromatin to DNA damage signaling. Cell Cycle 9: 930–936. 10.4161/cc.9.5.10931 20160506 PMC2901859

[2] Sun Y, Jiang X, Xu Y, Ayrapetov MK, Moreau LA, Whetstine JR, et al (2009) Histone H3 methylation links DNA damage detection to activation of the tumour suppressor Tip60. Nat Cell Biol 11: 1376–1382. 10.1038/ncb1982 19783983 PMC2783526

[3] Sykes SM, Mellert HS, Holbert MA, Li K, Marmorstein R, Lane WS, et al (2006) Acetylation of the p53 DNA-binding domain regulates apoptosis induction. Mol Cell 24: 841–851. 10.1016/j.molcel.2006.11.026 17189187 PMC1766330

[4] Tang Y, Luo J, Zhang W, Gu W (2006) Tip60-dependent acetylation of p53 modulates the decision between cell-cycle arrest and apoptosis. Mol Cell 24: 827–839. 10.1016/j.molcel.2006.11.021 17189186

[5] Squatrito M, Gorrini C, Amati B (2006) Tip60 in DNA damage response and growth control: many tricks in one HAT. Trends Cell Biol 16: 433–442. 10.1016/j.tcb.2006.07.007 16904321

[6] Hu Y, Fisher JB, Koprowski S, McAllister D, Kim MS, Lough J (2009) Homozygous disruption of the Tip60 gene causes early embryonic lethality. Dev Dyn 238: 2912–2921. 10.1002/dvdy.22110 19842187 PMC2801416

[7] Lough JW (2002) Transient expression of TIP60 protein during early chick heart development. Dev Dyn 223: 419–425. 10.1002/dvdy.10058 11891991

[8] Xiao G, Mao S, Baumgarten G, Serrano J, Jordan MC, Roos KP, et al (2001) Inducible activation of c-Myc in adult myocardium in vivo provokes cardiac myocyte hypertrophy and reactivation of DNA synthesis. Circ Res 89: 1122–1129. 11739276 10.1161/hh2401.100742

[9] Fisher JB, Kim MS, Blinka S, Ge ZD, Wan T, Duris C, et al (2012) Stress-induced cell-cycle activation in Tip60 haploinsufficient adult cardiomyocytes. PLoS One 7: e31569 10.1371/journal.pone.0031569 22348108 PMC3279378

[10] Sdek P, Zhao P, Wang Y, Huang CJ, Ko CY, Butler PC, et al (2011) Rb and p130 control cell cycle gene silencing to maintain the postmitotic phenotype in cardiac myocytes. J Cell Biol 194: 407–423. 10.1083/jcb.201012049 21825075 PMC3153646

[11] Heallen T, Morikawa Y, Leach J, Tao G, Willerson JT, Johnson RL, et al (2013) Hippo signaling impedes adult heart regeneration. Development 140: 4683–4690. 10.1242/dev.102798 24255096 PMC3833428

[12] Mahmoud AI, Canseco D, Xiao F, Sadek HA (2014) Cardiomyocyte cell cycle: Meis-ing something? Cell Cycle 13: 1057–1058. 10.4161/cc.28379 24603411 PMC4013154

[13] Ullman-Cullere MH, Foltz CJ (1999) Body condition scoring: a rapid and accurate method for assessing health status in mice. Lab Anim Sci 49: 319–323. 10403450

[14] Tybulewicz VL, Crawford CE, Jackson PK, Bronson RT, Mulligan RC (1991) Neonatal lethality and lymphopenia in mice with a homozygous disruption of the c-abl proto-oncogene. Cell 65: 1153–1163. 2065352 10.1016/0092-8674(91)90011-m

[15] Lee EC, Yu D, Martinez de Velasco J, Tessarollo L, Swing DA, Court DL, et al (2001) A highly efficient Escherichia coli-based chromosome engineering system adapted for recombinogenic targeting and subcloning of BAC DNA. Genomics 73: 56–65. 10.1006/geno.2000.6451 11352566

[16] Copeland NG, Jenkins NA, Court DL (2001) Recombineering: a powerful new tool for mouse functional genomics. Nat Rev Genet 2: 769–779. 10.1038/35093556 11584293

[17] Ng WA, Grupp IL, Subramaniam A, Robbins J (1991) Cardiac myosin heavy chain mRNA expression and myocardial function in the mouse heart. Circ Res 68: 1742–1750. 2036722 10.1161/01.res.68.6.1742

[18] Davis J, Maillet M, Miano JM, Molkentin JD (2012) Lost in transgenesis: a user's guide for genetically manipulating the mouse in cardiac research. Circ Res 111: 761–777. 10.1161/CIRCRESAHA.111.262717 22935533 PMC3466061

[19] Koitabashi N, Bedja D, Zaiman AL, Pinto YM, Zhang M, Gabrielson KL, et al (2009) Avoidance of transient cardiomyopathy in cardiomyocyte-targeted tamoxifen-induced MerCreMer gene deletion models. Circ Res 105: 12–15. 10.1161/CIRCRESAHA.109.198416 19520971 PMC2747596

[20] Banerjee I, Fuseler JW, Price RL, Borg TK, Baudino TA (2007) Determination of cell types and numbers during cardiac development in the neonatal and adult rat and mouse. Am J Physiol Heart Circ Physiol 293: H1883–1891. 10.1152/ajpheart.00514.2007 17604329

[21] Pinto AR, Ilinykh A, Ivey MJ, Kuwabara JT, D'Antoni ML, Debuque R, et al (2016) Revisiting Cardiac Cellular Composition. Circ Res 118: 400–409. 10.1161/CIRCRESAHA.115.307778 26635390 PMC4744092

[22] Xu Y, Liao R, Li N, Xiang R, Sun P (2014) Phosphorylation of Tip60 by p38alpha regulates p53-mediated PUMA induction and apoptosis in response to DNA damage. Oncotarget 5: 12555–12572. 10.18632/oncotarget.2717 25544752 PMC4350347

[23] Gogna R, Madan E, Khan M, Pati U, Kuppusamy P (2013) p53's choice of myocardial death or survival: Oxygen protects infarct myocardium by recruiting p53 on NOS3 promoter through regulation of p53-Lys(118) acetylation. EMBO Mol Med 5: 1662–1683. 10.1002/emmm.201202055 24096875 PMC3840484

[24] Tyteca S, Legube G, Trouche D (2006) To die or not to die: a HAT trick. Mol Cell 24: 807–808. 10.1016/j.molcel.2006.12.005 17189182

[25] Legube G, Linares LK, Tyteca S, Caron C, Scheffner M, Chevillard-Briet M, et al (2004) Role of the histone acetyl transferase Tip60 in the p53 pathway. J Biol Chem 279: 44825–44833. 10.1074/jbc.M407478200 15310756

[26] Sun Y, Jiang X, Chen S, Fernandes N, Price BD (2005) A role for the Tip60 histone acetyltransferase in the acetylation and activation of ATM. Proc Natl Acad Sci U S A 102: 13182–13187. 10.1073/pnas.0504211102 16141325 PMC1197271

[27] Berdougo E, Coleman H, Lee DH, Stainier DY, Yelon D (2003) Mutation of weak atrium/atrial myosin heavy chain disrupts atrial function and influences ventricular morphogenesis in zebrafish. Development 130: 6121–6129. 10.1242/dev.00838 14573521

[28] Huang C, Sheikh F, Hollander M, Cai C, Becker D, Chu PH, et al (2003) Embryonic atrial function is essential for mouse embryogenesis, cardiac morphogenesis and angiogenesis. Development 130: 6111–6119. 10.1242/dev.00831 14573518

[29] van Berlo JH, Molkentin JD (2014) An emerging consensus on cardiac regeneration. Nat Med 20: 1386–1393. 10.1038/nm.3764 25473919 PMC4418535

[30] Mahmoud AI, Kocabas F, Muralidhar SA, Kimura W, Koura AS, Thet S, et al (2013) Meis1 regulates postnatal cardiomyocyte cell cycle arrest. Nature 497: 249–253. 10.1038/nature12054 23594737 PMC4159712

[31] Gorrini C, Squatrito M, Luise C, Syed N, Perna D, Wark L, et al (2007) Tip60 is a haplo-insufficient tumour suppressor required for an oncogene-induced DNA damage response. Nature 448: 1063–1067. 10.1038/nature06055 17728759

[32] Ahuja P, Perriard E, Perriard JC, Ehler E (2004) Sequential myofibrillar breakdown accompanies mitotic division of mammalian cardiomyocytes. J Cell Sci 117: 3295–3306. 10.1242/jcs.01159 15226401

[33] D'Uva G, Aharonov A, Lauriola M, Kain D, Yahalom-Ronen Y, Carvalho S, et al (2015) ERBB2 triggers mammalian heart regeneration by promoting cardiomyocyte dedifferentiation and proliferation. Nat Cell Biol 17: 627–638. 10.1038/ncb3149 25848746

[34] Szibor M, Poling J, Warnecke H, Kubin T, Braun T (2014) Remodeling and dedifferentiation of adult cardiomyocytes during disease and regeneration. Cell Mol Life Sci 71: 1907–1916. 10.1007/s00018-013-1535-6 24322910 PMC11113405

[35] Kardami E, Dang X, Iacobas DA, Nickel BE, Jeyaraman M, Srisakuldee W, et al (2007) The role of connexins in controlling cell growth and gene expression. Prog Biophys Mol Biol 94: 245–264. 10.1016/j.pbiomolbio.2007.03.009 17462721

[36] Jeyaraman MM, Fandrich RR, Kardami E (2013) Together and apart: inhibition of DNA synthesis by connexin-43 and its relationship to transforming growth factor beta. Front Pharmacol 4: 90 10.3389/fphar.2013.00090 23882217 PMC3715720

[37] Severs NJ, Coppen SR, Dupont E, Yeh HI, Ko YS, Matsushita T (2004) Gap junction alterations in human cardiac disease. Cardiovasc Res 62: 368–377. 10.1016/j.cardiores.2003.12.007 15094356

[38] Puente BN, Kimura W, Muralidhar SA, Moon J, Amatruda JF, Phelps KL, et al (2014) The oxygen-rich postnatal environment induces cardiomyocyte cell-cycle arrest through DNA damage response. Cell 157: 565–579. 10.1016/j.cell.2014.03.032 24766806 PMC4104514

