# Supplementary figures and images for: Depletion of Tip60 from In Vivo Cardiomyocytes Increases Myocyte Density, Followed by Cardiac Dysfunction, Myocyte Fallout and Lethality
Source: PLoS One. 2016 Oct 21;11(10):e0164855. doi: 10.1371/journal.pone.0164855 (PMC5074524; doi:10.1371/journal.pone.0164855)

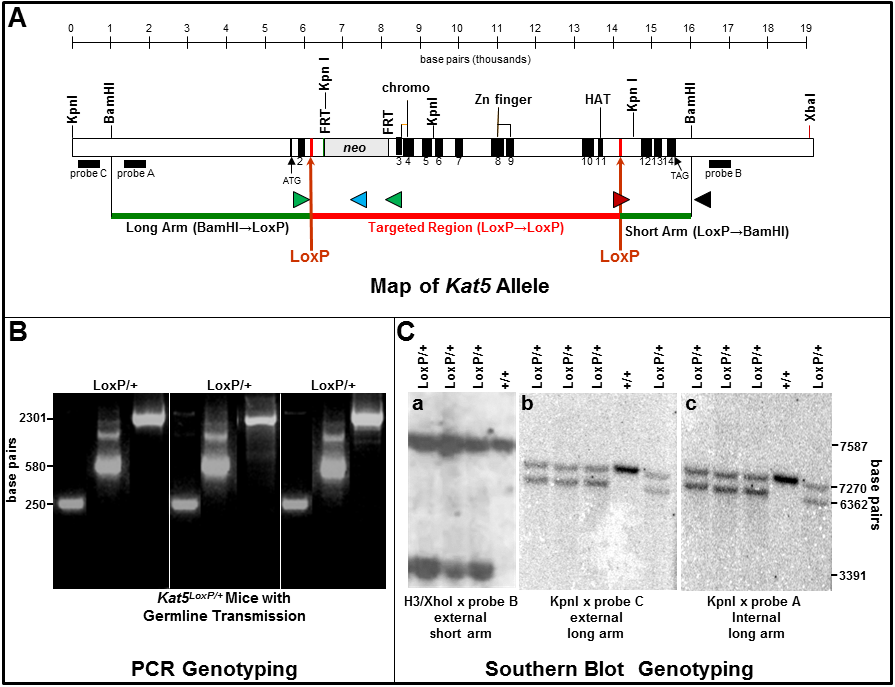

Supplement: S1 Fig — The targeting vector (Panel A) was prepared by recombineering to introduce LoxP sites into introns 2 and 11 of the Kat5 gene. This permits Cre-recombinase-mediated excision of exons 3–11, which consists of 71% of the exon structure including the acetyltransferase domain. Correctly targeted ESCs (line V6.5) were injected into C57Bl6 blastocysts, generating 100% chimeric males that transmitted the floxed allele via germline, providing the genotype Kat5LoxP(FRTneoFRT)/+. The neomycin phosphotransferase (neo) gene in intron 2, which was flanked by FRT sites, was removed by mating with a mouse expressing the Flp recombinase transgene. Kat5LoxP/- mice, obtained by mating Kat5LoxP/LoxP mice with Kat5+/- mice, were used for all experiments reported here because this necessitates deletion of only one allele to maximally reduce Tip60 protein levels. Panel A, Restriction Map of the LoxP-targeted Kat5 allele. Bars denote Southern blot probes A-D. Arrowheads denote PCR primer sites. Panel B, PCR Genotyping: The primer pair denoted by green arrowheads (A) amplifies a 250 bp band of the WT allele, which does not contain the previously removed Neo cassette. The 5’ green + 3’ blue (in neo) arrowhead pair in A amplifies 580 bp of the LoxP-modified allele. The red-black arrowhead pair amplifies 2,301 bp of the LoxP-modified allele (note that the red primer anneals to the LoxP locus). Panel C, Southern Blot Genotyping. a, double digest with Hind III (H3) and XhoI (within the LoxP site) followed by hybridization with probe B reveals a 3391 band diagnostic of the floxed allele, and a 7,587 band diagnostic of the WT allele; b, digestion with KpnI and hybridization with probe C yields a 7270 bp band from the WT allele, and a 6362 bp fragment created by a KpnI site within the neo cassette of the floxed allele; c is the same as b except that hybridization was with probe A, an internal probe used to ensure that the targeting vector did not randomly insert into a non-endogenous target(s). (TIF [file pone.0164855.s001.tif]

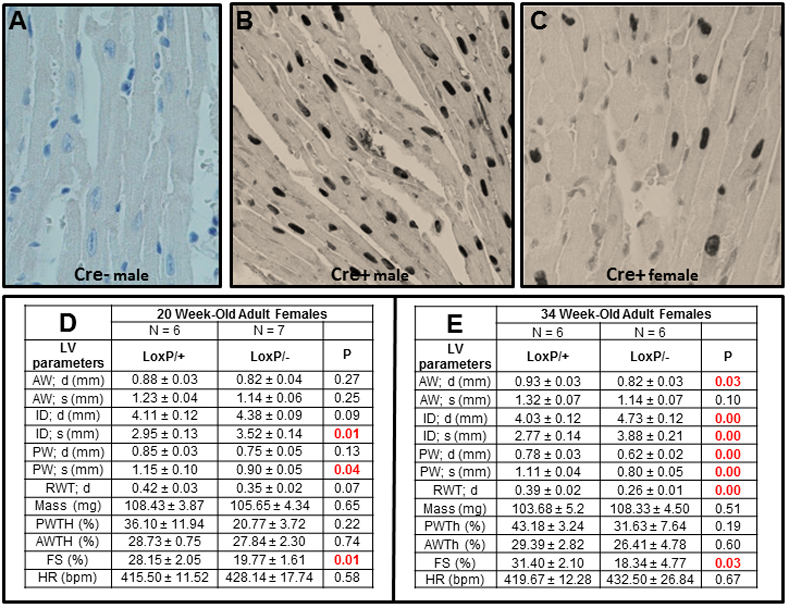

Supplement: S2 Fig — A-C, typical immunohistochemical staining pattern of Cre-recombinase showing labeled nuclei in approximately one-half of female (C), and in 100% of male (B) CMs. This phenomenon is presumably caused by presence of the Myh6-Cre transgene on the X chromosome, half of which randomly undergo X-inactivation in female mice. D-E, Echocardiographic determinations performed on female hearts showing that although survival is not compromised up to 34 weeks of age, systolic function is affected by week 20. P-values were calculated using Student’s T-Test (two-tailed; unpaired). All CMs contained the Myh6-Cre transgene. (TIF) [file pone.0164855.s002.tif]

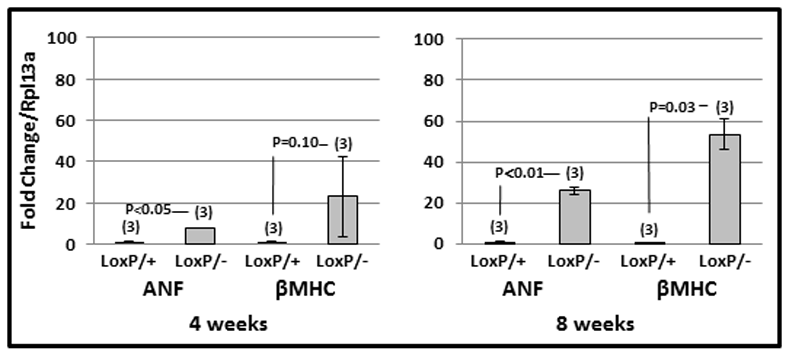

Supplement: S3 Fig — qPCR showing progressively increased expression of ANF and β-MHC in Tip60-depleted hearts of 4 and 8 week-old male mice. P-values were calculated using Student’s T-Test (two-tailed). All CMs contained the Myh6-Cre transgene. (TIF) [file pone.0164855.s003.tif]

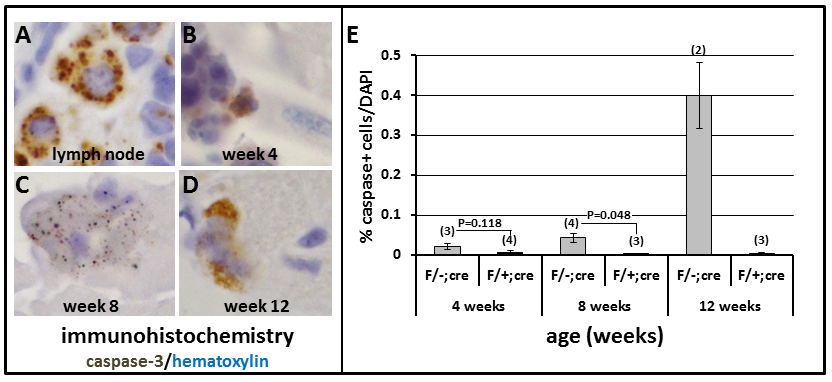

Supplement: S4 Fig — Cells in panels A-D were photographed at 60x, revealing cytoplasmic granular staining of caspase-3 (Biocare cp229b) in Kat5LoxP/-;Myh6-Cre (F/-;cre) hearts at each indicated age. Panel A is a positive control (mouse lymph node). In E, entire sections from each heart were scanned at 60x, during which all caspase-3-positive cells in each section were manually enumerated, followed by normalization to the total number of nuclei assessed by automated scanning of DAPI-stained immediately adjacent sections. F/-;cre denotes hearts from Kat5LoxP/-;Myh6-Cre mice; F/+;cre denotes hearts from Kat5LoxP/+;Myh6-Cre mice. The number above each bar indicates the number of mouse hearts that were evaluated. Vertical lines = ±SEM. P-values were calculated using Student’s T-Test (two-tailed). All CMs contained the Myh6-Cre transgene. (TIF) [file pone.0164855.s004.tif]
