# Supplementary material for: Depletion of Tip60 from In Vivo Cardiomyocytes Increases Myocyte Density, Followed by Cardiac Dysfunction, Myocyte Fallout and Lethality
Source: PLoS One. 2016 Oct 21;11(10):e0164855. doi: 10.1371/journal.pone.0164855 (PMC5074524; doi:10.1371/journal.pone.0164855)
Supplement: S1 File — Details regarding animal care and echocardiography. (DOCX) [file pone.0164855.s005.docx]

**Supporting Information**

**Animal Protocols**

This investigation adhered to the National Institutes of Health (NIH) Guide for the Care and Use of Laboratory Animals (NIH Pub. Nos. 85-23, Revised 1996). All protocols described in the corresponding author’s Animal Use Application (AUA #00000225 entitled “Tip60 and Heart Development”) were approved by the Medical College of Wisconsin’s Institutional Animal Care and Use Committee (IACUC). The Medical College of Wisconsin has an Animal Welfare Assurance status from the Office of Laboratory Welfare (A3102-01).

**Humane Endpoints**

This study utilized genetic disruption of the *Kat5* gene from cardiomyocytes to assess the importance of Tip60 function in the heart.  We observed that Tip60 depletion caused mortality between 8-12 weeks of age. Because this outcome was not anticipated, it was not reviewed and approved by the animal ethics committee as part of our IACUC-approved protocol.  Despite daily monitoring to identify humane endpoints based on BCS (body condition scoring, [[1](#_ENREF_1)]) criteria, neither a BCS score of “3” in a single category nor an aggregate score of “7” across categories was observed prior to death. All male mice (approximately 25 in this study) bearing the *Kat5^LoxP/-;Myh6-Cre^* genotype spontaneously died at approximately 12 weeks of age.  [Note: BCS criteria that were monitored included discomfort (assessed by hunching with disheveled fur), lethargy, rapid weight loss, labored breathing, and behavioral response to external stimuli.]

**Euthanasia**

Mice were euthanized if they reached the humane endpoint criteria described above, or if they were of incorrect genotype or gender (identified upon weaning). Mice were euthanized by CO_2_ asphyxiation using continuous flow in an enclosed non-opaque chamber. CO_2_ was delivered at a flow rate of ~20% of the chamber volume per minute. Animals remained in the chamber for at least 5 minutes, and were removed only after there was absence of respiratory activity or a detectable heart beat. After removal from the chamber, cervical dislocation was performed, or an incision was made into the thorax to ensure death.

**Frequency of Monitoring**

Mice were monitored daily by laboratory staff. In addition, animal housing staff monitored the mice a minimum of once per day. All unexpected deaths were attributed to Tip60 depletion in cardiomyocytes with death occurring suddenly between 8 and 12 weeks of age. The cause of death was uncertain, but appeared to be related to heart failure or arrhythmia. Animals that achieved a BCS score of 3 in one category and an aggregate score of 7 across categories were immediately euthanized and considered a mortality data point at that time.

**Administration of Analgesics & Anesthetics**

These interventions were not required to minimize animal suffering and distress at any point during the study, except during echocardiography (described below).

**Animal Husbandry, Housing, Diet, & Care**

These were provided by staff employed by the Medical College of Wisconsin Biomedical Resource Center. Mice were housed in Zytem bio-contained micro-isolated cages within a wire rack to provide water. No more than five mice were housed per cage. Mice were fed standard Harlan rodent pellets. Each cage was provided with nesting shavings and Enviro-Dri® nesting material. For breeding, one or two females were housed with one male stud; each female mouse was allowed to produce one litter. Upon attaining three weeks of age, pups were weaned, at which time they were genotyped, sexed and separated by gender into different cages, each of which was provided a water bottle and crushed food.

1. **Number & Gender of Mice**
2. Approximately 384 mice were employed in this study. This number was required to satisfy the experimentally mandated requirements to utilize (i) only male mice and (ii) approximately equal numbers of male mice bearing the genotypes *Kat5^LoxP/-;Myh6-Cre^* and *Kat5^LoxP/+;Myh6-Cre^* for comparison; breeding of the these genotypes was only ~25% efficient.

**Echocardiography**

Transthoracic echocardiography was performed using a VisualSonics Vevo 770 ultrasound system. For this procedure, anesthesia was induced by placing mice into an induction chamber containing 2% isoflurane. Once anesthetized, mice were transferred to a heated platform and anesthesia was maintained at a concentration of 1-2% isoflurane using a nose cone. For ultrasound measurements, mice were taped to a warmed mouse handling table (VisualSonics) that obtains ECG recordings. Hair was removed from the chest by shaving and then with a topical depilatory agent. Warmed ultrasound gel was applied to the chest wall, and the probe was advanced to obtain recordings using parasternal short and parasternal long axis views with very gentle pressure (so as not to compress the chest of the animal and to obtain optimal images). Once images were obtained, mice were removed from the anesthetic agent and allowed to recover in their cages. After the procedure, mice were observed for at least 1 h to ensure recovery before returning to housing in the Biomedical Resource Center. These studies were performed in the Ultrasound Core Suite located in the Cardiovascular Center under supervision of Dr. Tina Wan. The suite is equipped to remove anesthetic exhaust by connection to a vacuum system.

**Supporting Reference**

1. Ullman-Cullere MH, Foltz CJ (1999) Body condition scoring: a rapid and accurate method for assessing health status in mice. Lab Anim Sci 49: 319-323.
